# Improving Antibiotic Use in Nursing Homes by Infection Prevention and Control and Antibiotic Stewardship (IMAGINE): Protocol for a Before-and-After Intervention and Implementation Study

**DOI:** 10.2196/60099

**Published:** 2024-09-16

**Authors:** Ana García-Sangenís, Daniela Modena, Jette Nygaard Jensen, Athina Chalkidou, Valeria S Antsupova, Tina Marloth, Anna Marie Theut, Beatriz González López-Valcárcel, Fabiana Raynal, Laura Vallejo-Torres, Jesper Lykkegaard, Malene Plejdrup Hansen, Jens Søndergaard, Jonas Kanstrup Olsen, Anders Munck, András Balint, Ria Benko, Davorina Petek, Nina Sodja, Anna Kowalczyk, Maciej Godycki-Cwirko, Helena Glasová, Jozef Glasa, Ruta Radzeviciene Jurgute, Lina Jaruseviciene, Christos Lionis, Marilena Anastasaki, Agapi Angelaki, Elena Petelos, Laura Alvarez, Marta Ricart, Sergi Briones, Georg Ruppe, Ramon Monfà, Anders Bjerrum, Carl Llor

**Affiliations:** 1 Fundació Institut Universitari per a la Recerca a l'Atenció Primària de Salut Jordi Gol Barcelona Spain; 2 Department of Clinical Microbiology Copenhagen University Hospital – Herlev and Gentofte Copenhagen Denmark; 3 Department of Quantitative Methods in Economics and Management University of Las Palmas de Gran Canaria Las Palmas de Gran Canaria Spain; 4 Research Unit for General Practice Department of Public Health University of Southern Denmark Odense Denmark; 5 Center for General Practice Aalborg University Aalborg Denmark; 6 Szeged Autumns Nursing Home Szeged Hungary; 7 University of Szeged Szeged Hungary; 8 Department of Family Medicine University of Ljubljana Ljubljana Slovenia; 9 Centre for Family and Community Medicine Faculty of Health Sciences Medical University of Lodz Lodz Poland; 10 Department of Clinical Pharmacology Faculty of Medicine Slovak Medical University Bratislava Slovakia; 11 Family Medicine Department Lithuanian University of Health Sciences Kaunas Lithuania; 12 Clinic of Social and Family Medicine School of Medicine University of Crete Heraklion Greece; 13 Spanish Society for Family and Community Medicine Barcelona Spain; 14 European Union of Geriatric Medicine Society Vienna Austria; 15 Institut Català de la Salut Via Roma Health Centre Barcelona Spain

**Keywords:** antimicrobial stewardship, medical audit, hygiene, antibacterial agents, quality improvement, nursing homes, health personnel, drug resistance, microbial, frail elderly

## Abstract

**Background:**

Despite the extensive use of antibiotics and the growing challenge of antimicrobial resistance, there has been a lack of substantial initiatives aimed at diminishing the prevalence of infections in nursing homes and enhancing the detection of urinary tract infections (UTIs).

**Objective:**

This study aims to systematize and enhance efforts to prevent health care–associated infections, mainly UTIs and reduce antibiotic inappropriateness by implementing a multifaceted intervention targeting health care professionals in nursing homes.

**Methods:**

A before-and-after intervention study carried out in a minimum of 10 nursing homes in each of the 8 European participating countries (Denmark, Greece, Hungary, Lithuania, Poland, Slovakia, Slovenia, and Spain). A team of 4 professionals consisting of nurses, doctors, health care assistants, or health care helpers are actively involved in each nursing home. Over the initial 3-month period, professionals in each nursing home are registering information on UTIs as well as infection and prevention control measures by means of the Audit Project Odense method. The audit will be repeated after implementing a multifaceted intervention. The intervention will consist of feedback and discussion of the results from the first registration, training on the implementation of infection and prevention control techniques provided by experts, appropriateness of the diagnostic approach and antibiotic prescribing for UTIs, and provision of information materials on infection control and antimicrobial stewardship targeted to staff, residents, and relatives. We will compare the pre- and postintervention audit results using chi-square test for prescription appropriateness and Student *t* test for implemented hygiene elements.

**Results:**

A total of 109 nursing homes have participated in the pilot study and the first registration audit. The results of the first audit registration are expected to be published in autumn of 2024. The final results will be published by the end of 2025.

**Conclusions:**

This is a European Union–funded project aimed at contributing to the battle against antimicrobial resistance through improvement of the quality of management of common infections based on evidence-based interventions tailored to the nursing home setting and a diverse range of professionals. We expect the intervention to result in a significant increase in the number of hygiene activities implemented by health care providers and residents. Additionally, we anticipate a marked reduction in the number of inappropriately managed UTIs, as well as a substantial decrease in the overall incidence of infections following the intervention.

**International Registered Report Identifier (IRRID):**

DERR1-10.2196/60099

## Introduction

Life expectancy has been steadily increasing in the European Union. Population projections estimate that by 2050 the old-age dependency ratio, calculated as the number of individuals older than 65 years per 100 people of working age, will reach 50% [1]. The rise in the ageing population and the pressure on the health care provision systems across European countries has led to reductions in hospital beds and more patient care being provided in long-term care facilities [2]. In 2019, there were an estimated 2.9 million residents in such facilities, corresponding to approximately 0.7% of the total population in the European Union [3].

Residents in nursing homes are more prone to frequent and severe infections [4]. In general, infections are highly prevalent among older people, especially those living in nursing homes, as they are more vulnerable to urinary tract infections (UTIs), respiratory tract infections, and acute bacterial skin- and soft-tissue infections [5]. Although recent estimates are lacking, approximately 2 million infections occur in US nursing homes each year [6]. Residents in care homes are particularly susceptible to infections due to factors such as immunosenescence, multiple comorbidities, functional impairment, the use of indwelling devices such as catheters and feeding tubes, close living proximity, recent acute care admissions, and frequent contact with nursing staff and medical equipment [7-10]. Microorganisms can be found on residents, relatives, and friends, on surfaces in the facility, and even on the hands or gloves of health care professionals (HCP) and medical equipment. Without appropriate and adequate cleaning and disinfection methods, the germs may spread to other residents and the environment. In younger healthy individuals, the immune system fights off germs and prevents infection; however, the declining ability of the body and immune systems to resist germs makes older people more susceptible to infection and they can become infected more easily [11,12].

Antibiotic overprescribing for infectious diseases is high in nursing homes. In a recent project on the prevalence of antimicrobial agents in 3052 long-term care facilities in 24 European Union countries in 2016-2017, 4.9% of the residents had received at least 1 antimicrobial agent on the day of the survey [13]. This study also showed that for a 1% increase in the proportion of residents older than 85 years, the prevalence of antimicrobial use increased by 5%. In addition, approximately 30% of the antibiotics prescribed were given prophylactically, and most were given inappropriately, with a wide variation across European countries. Furthermore, the urinary tract was the most common body site for which antimicrobials are prescribed, with nearly 50% of the cases, and according to the authors, at least 60% of these antimicrobials prescribed for UTI were inappropriate, leading to a spread of multidrug-resistant uropathogens [13]. As a result, Watch and Reserve broad-spectrum antibiotics, as defined by the World Health Organization Access, Watch, Reserve classification [14], are commonly given to residents with these infections, perpetuating the problem of antimicrobial resistance (AMR), which is accompanied with higher morbidity, mortality, health, and intersectoral and societal costs [15].

In nursing homes, antibiotic prescribing decisions are known to be complex and influenced by many social and organizational factors [16-18]. Overdiagnosis of common infections largely explains antibiotic overuse for these infections. A review of 4 randomized controlled trials of interventions aimed at improving the diagnosis of UTIs in nursing home residents found that, despite being heterogeneous, the interventions used were to some extent successful, but there was no evidence that an intervention was feasible or sustainable following trial completion [19]. Several decision tools have been developed for the diagnosis of UTIs in nursing home residents to assist clinicians in identifying symptomatic UTI [20,21]. The overarching message is that there are no precise diagnostic rules for identifying UTI among elderly individuals in nursing homes. In general, there is agreement that nonspecific symptoms alone, such as changes in mental or functional status or changes in urine characteristics, including bad smell, color, or sediment, should not be used to diagnose UTI and that a diagnosis of UTI in nursing home residents should not be made on urinalysis or urine culture without UTI-specific symptoms.

Antibiotic-resistant bacteria have the potential to spread between individuals through direct or indirect contact. Therefore, the implementation of infection prevention and control (IPC) strategies plays a crucial role in reducing the risk of infection [22]. These strategies not only contribute to a decrease in the number of infections but also effectively and efficiently reduce antimicrobial consumption, thereby limiting opportunities for misuse [23]. It is essential to emphasize the significance of infection prevention, biosecurity measures, and control practices in managing all infectious microorganisms, as they minimize the reliance on antimicrobials and, consequently, hinder the development and spread of resistance [24]. Unfortunately, there is a general lack of IPC measures and antimicrobial stewardship (AMS) programs in nursing homes across Europe [13]. In addition, most countries exhibit weak interprofessional collaboration and inadequate integration of health and social care delivery, leading to increased mortality, as evidenced during the COVID-19 pandemic [25,26].

In summary, in the effort to urgently decelerate the development of AMR, there is a need for evidence-based nursing home-focused AMS policies and IPC strategies aimed at preventing and controlling the spread of infections, as well as promoting appropriate antibiotic use across health care settings. Our objective is to assess the impact of a multifaceted intervention aimed at nursing home HCPs on preventing common infections, particularly UTIs. Additionally, we aim to evaluate the effectiveness of this intervention in reducing the percentage of inappropriate antibiotic use for common infections. By targeting HCPs with comprehensive training and resources, we hope to improve infection control practices and promote more judicious use of antibiotics, ultimately enhancing the quality of care for nursing home residents.

## Methods

### Study Design

This is a before-and-after intervention study in HCPs working in nursing homes. We are using the Audit Project Odense methodology [27], as the strategy to implement the multifaceted actions suggested in point 4.2 of the European Union’s AMR Guidelines for long-term care facilities states [23]: “establish a multi-faceted approach which includes elements such as education of nursing and medical staff, audits of antimicrobial use, feedback to the prescribers, and targeting identified areas of antimicrobial overuse and misuse.” The improving antibiotic use in nursing homes by infection prevention and control and antibiotic stewardship (IMAGINE) project was initiated in January 2023 and is planned to finish in December 2025. The consortium includes 12 partners from 9 countries, as indicated in Table 1.

**Table 1:** Partners in IMAGINE^a^.

Participant organization name	Abbreviation	Role
Institut Català de la Salut and Fundació Institut Universitari per a la Recerca a l'Atenció Primària de Salut Jordi Gol, Spain	ICS or IDIAP	Coordinator of the project. Spanish coordinator
The Capital Region of Denmark	CAP	Preparation of materials for intervention. Danish coordinator
Research Unit for General Practice Odense, Denmark	RUPO	Audit Project Odense methodology
Szeged Autumns Nursing Home, Hungary	SAN	Hungarian coordinator
University of Ljubliana, Slovenia	UOL	Slovenian coordinator
Medical University of Lodz, Poland	MUL	Polish coordinator
Slovak Medical University in Bratislava, Slovakia	SMU	Slovak coordinator
Ltd Mano Seimos Gydytojas (My Family Doctor)	FDC	Lithuanian coordinator
University of Crete, Greece	UOC	Greek coordinator
University of Las Palmas de Gran Canaria and Fundación Canaria Parque Científico Tecnológico	ULPGC or FCPCT	Analysis and evaluation of the results
Spanish Society for Family and Community Medicine	SEMFYC	Dissemination and training in communication skills
European Union of Geriatric Medicine Society	EUGMS	In charge of dissemination of results

^a^IMAGINE: improving antibiotic use in nursing homes by infection prevention and control and antibiotic stewardship.

### Aims

The primary objectives of this study (IMAGINE project) are aimed at increasing the IPC elements implemented by 50% and decreasing the incidence of inappropriate antibiotic consumption for UTIs by 40%, with a subsequent 20% reduction in the incidence of UTIs. Different secondary objectives are planned: (1) reduction in inappropriate use of urinary catheters in nursing home residents; (2) reduction in the percentage of inappropriate antibiotic use for the prophylaxis of UTIs; (3) reduction in the number of Watch and Reserve antibiotics from the World Health Organization Access, Watch, Reserve classification; (4) improvement in the satisfaction of nursing home HCPs, residents, and relatives; (5) reduction in the incidence of health care–associated infections treated with antibiotics others than UTI; and (6) reduction in the percentage of hospital admissions.

### Setting

We are carrying out interventions in nursing homes in 8 European countries: Denmark, Greece, Hungary, Lithuania, Poland, Slovakia, Slovenia, and Spain. In each country, at least 10 nursing homes participate and a team of 4 HCPs in each nursing home are actively involved. Different types of professionals are involved in the health care of residents in the nursing homes: registered nurses, doctors that can be external or working in the facility part-time, health care assistants, or health care helpers. The participating nursing homes select the HCPs of their setting who actively take part in the study. These HCPs will, in turn, become trainers in their own nursing home after the intervention meetings.

### Project Methodology

**Figure 1:**
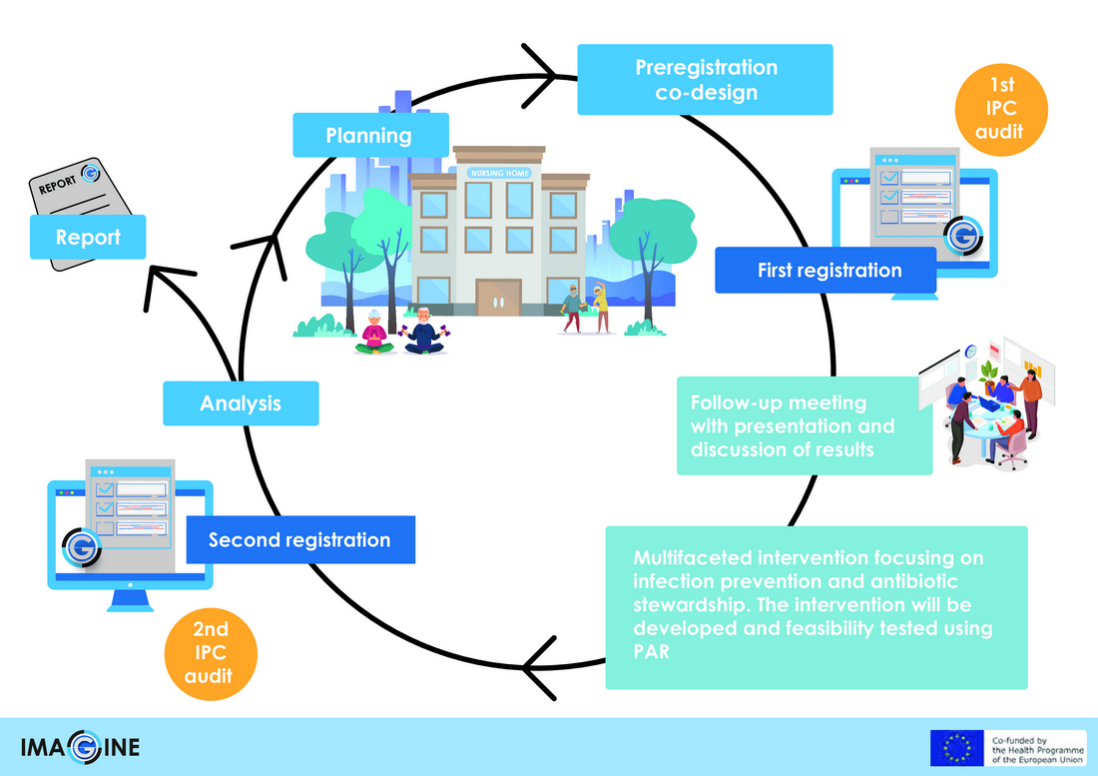
Audit Project Odense cycle in the IMAGINE (improving antibiotic use in nursing homes by infection prevention and control and antibiotic stewardship) project. IPC: infection prevention and control; PAR: participatory action research.

A point prevalence audit is scheduled to assess the outcomes of the multifaceted intervention [27]. The audit is conducted in 2 separate 3-month periods: one before the intervention and another after. The initial registration occurred from February 2024 to April 2024, prior to the intervention, while the second registration will take place from February 2025 to April 2025, after the intervention (Figure 1). Throughout the audit, HCPs record anonymized information on UTIs antibiotic prescriptions on a chart. Sometime during the audit registration, participants are asked to also register the IPC measures in place in the nursing homes. Between the first and second audits, participants will attend a multifaceted intervention aimed at improving the prevention and management of infections in nursing homes (November 2024). The intervention and the data collection sheets are specifically designed for the study, based on preliminary work conducted in 2023.

### Preliminary Study: Context Analysis

A preliminary study was conducted to identify potential areas for improvement in nursing homes across the 8 participating countries. The study involved a context analysis, which included interviews with HCPs working in nursing homes. These interviews focused on IPC practices, as well as antibiotic management. The aim is to explore the factors that contribute to the spread of infections within the target organizations.

Qualitative analysis was used to incorporate the perspectives of various nursing home HCPs in the development of educational materials. These materials facilitate communication between HCPs, residents, and their relatives when discussing the necessity and appropriate use of antibiotics for common infections. The analysis also helps identify any gaps in information that need to be addressed during the multifaceted intervention. Local coordinators within the 8 target countries of the IMAGINE project have contacted nursing home HCPs, who serve as the panel of experts for prioritizing areas of improvement. Their expertise guides the project’s focus on enhancing practices in nursing homes.

### Data Collection

**Figure 2:**
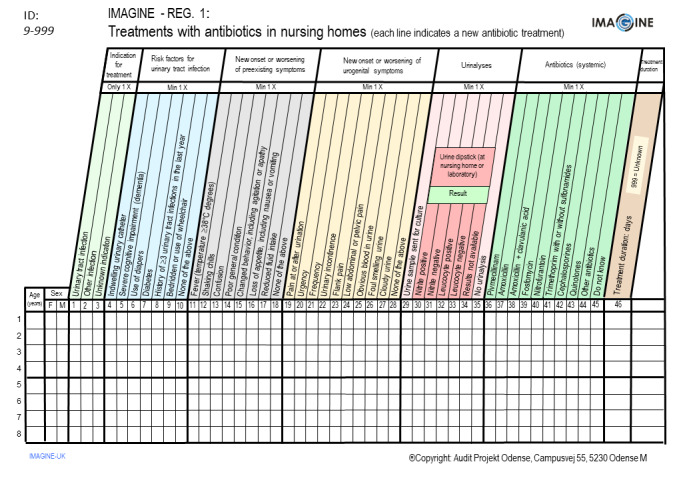
Template used in the IMAGINE (improving antibiotic use in nursing homes by infection prevention and control and antibiotic stewardship) project for patients receiving antibiotics. Subject to subsequent changes that may arise during the pilot study.

A specific registration chart has been developed considering the characteristics and the quality improvement areas identified during the preparatory study performed in 2023. During the audit period, the participants are using the Audit Project Odense registration chart developed to record all the antibiotic prescriptions made in the nursing home. At most, 10 main groups and a maximum of 45 variables are used to describe the topic being investigated [27]. The main groups are lined up in a logical way, for example, focus of infection, symptoms, indwelling urinary catheter, diagnostic tests, treatment, and referral or not. The variables are exhaustive (include all possibilities) and exclusive (no overlapping), and all are in the same logic plan. One line is filled in for each case. As a rule, only ticks are allowed (no writing). At least 1 tick per main group is needed. Figure 2 depicts the definitive template used by IMAGINE. A short instruction sheet (maximum 1 page) has been provided for all participants. The instruction sheet specifies the registration period, the inclusion and exclusion criteria for the cases and briefly explains the content of each main group. Information about where to return the completed charts was given. A local contact person from the national teams is available for questions from the participants [27]. A pilot test of the chart was performed between October 2023 and November 2023 with 3 to 5 nursing home HCPs per country to ensure that the content of the registration chart was easily understood and to confirm that enough cases are available.

### Multifaceted Intervention

Interventions that are expected to have the highest likelihood of success appear to align with the constructs of the normalization process theory, which explain the mechanisms of implementation [28]. These constructs include coherence (making sense of interventions), cognitive participation (engagement with the intervention), collective actions (enabling the intervention to take place), and reflexive monitoring (evaluating the costs and benefits). In the IMAGINE project, the multifaceted intervention aims to address all dimensions of the normalization process theory. It will achieve this by facilitating open discussions regarding variations in practice behaviors and by strengthening communication between HCPs, residents, and their families. Through these efforts, the intervention seeks to enhance the understanding and acceptance of the intervention among all stakeholders involved.

The multifaceted intervention will be composed by face-to-face and web-based activities, the latter in an asynchronous manner, enabling nursing home HCPs to engage in these activities at their own convenience. The main part of the training will be a 2-day intervention program consisting of (1) presentation of the first audit results and discussion with the rest of their colleagues in their respective countries, allowing self-reflection of their performance and discussing quality indicators associated with their practice; (2) AMR and IPC elements, with the participation of national experts; (3) discussion on AMS on how to improve the diagnosis of UTIs and the role of antibiotics for these infections; and (4) discussion of best practice case studies and practical issues.

The courses will be based on active discussion among participants, with the use of videos, leaflets, digital material, and role-playing, allowing fruitful discussion. This intervention will be recorded and adapted to a web-based environment. All material created for the intervention will be used to achieve three main objectives: (1) reinforcing the training of the HCPs, allowing them to revisit concepts whenever they need them; (2) training of the rest of the nursing home staff on IPC measures and AMS; and (3) after the end of the project, training of HCPs in other nursing homes in the country.

The intervention will be tailored to the nursing home setting. Achieving a significant behavioral change among multiple HCPs is essential for improving antibiotic prescribing in this population. To accomplish this, we will use the participatory action research methodology, which actively involves HCPs in the implementation of IPC and AMS interventions that are customized to their specific health care settings. This approach takes into account the local barriers and facilitators that may affect the successful implementation of these interventions [29]. The primary objective of the participatory action research is to generate practical knowledge that can be readily applied in local practice. By using this methodology, we aim to gain a better understanding of the complex decision-making process related to IPC practices and suspected health care–associated infections among frail elderly residents in nursing homes. It is important to consider the diverse elderly care settings across the various target countries, and participatory action research will help us navigate this heterogeneity [30].

National HCPs and stakeholders will participate to identify factors in the nursing home setting that contribute to increased risk of spread of infection and to determine national characteristics of nursing home management, diagnosis, and antibiotic prescription practices for nursing home residents and elderly patients with symptoms of infection. They will also work together to identify quality improvement areas related to hygiene, infection prevention, and inappropriate use of antibiotics; define priorities to be included when developing the educational material that will be used for facilitating communication between HCPs, residents, and relatives; and define an algorithm to be used for analyzing antibiotic inappropriateness in the different types of health care–associated infections.

### Data Analysis Plan

The data collected in the specific registration charts have a primary function at the individual level of health professionals, that is, helping them to improve their practice by reconsidering their treatment choices considering clinical evidence, guidelines, and professional consensus [31]. The second function is to analyze the data to evaluate the effect of the intervention. In each audit, data are recorded for all antibiotics prescribed during the 3-month period. We aim to record a minimum of 50 cases in each of the nursing homes recruited (at least 100 nursing homes), yielding an approximate number of 5000 cases in each registration period.

We will apply the chi-square test to determine the frequencies and appropriateness of prescriptions before and after the intervention and Student *t* test for the number of hygiene elements implemented in the nursing homes to test the null hypothesis of no effect of the interventions. To measure the potential reduction in UTIs, we will assume that antibiotics are always prescribed for these infections and every antibiotic is associated with a new episode of UTI [32]. The number of hygiene elements implemented will be compared with a threshold that will be defined during the participatory action research analysis. The appropriateness of antibiotic prescriptions will be defined according to an algorithm based on symptoms and patient characteristics that will be developed at the start of the project. We will also estimate the effect of the interventions on inappropriate prescriptions for UTIs using multilevel logistic regression models with 2 levels: cases and HCP participants, to allow for the fact that patients belonging to the same HCP are not independent of each other [33]. Statistical significance will be considered with *P*<.05. The data will be analyzed using the current version of the Stata statistical program (version 18, StataCorp LLC).

### Data Management

The collection of the audit data is performed on paper. The registration templates will be stored securely in in each participating nursing home and will be accessible only by study staff and authorized personnel. Charts will be transferred electronically to the Audit Project Odense service provider to be entered in a database [31]. Data from residents will be anonymously collected from the beginning and no identifiable or personal information will be gathered. Nursing homes will be identified by a code. Qualitative interviews will be audio recorded, transcribed, and translated, and any identifiable information will be deleted in the process. Translations will be transferred securely to the organization in charge of analyzing the results. Anonymized data from the survey will be stored for a minimum of 5 years after publication of the results. Anonymized project data will be shared for common analyses and presentation to the scientific community through publications and conferences.

### Ethical Considerations

The study is conducted in accordance with the protocol, the Declaration of Helsinki, the principles of Good Clinical Practice, The General Data Protection Regulation (of the European Union) 2016/679 and the Human Research Act as well as other locally relevant regulations. Appropriate regulatory or ethical approval has been sought in each of the countries taking part in the study, and all study procedures started after gaining approval on the basis of the master protocol, translated where necessary to local language. In Spain, the coordinating country, the study protocol was approved by the Ethics Committee of Institut Universitari d’Investigació en Atenció Primària (IDIAP Jordi Gol), Institute of Research in Primary Health Care (23/080-P).

Nursing homes have been contacted by local coordinators that have explained the study and handed over the information, giving the nursing home time to read it and ask any questions. Informed consent was obtained from all participants. HCPs are participating voluntarily in this quality improvement project. No personnel data are collected from either HCPs or patients. A nursing home can withdraw from the project at any time without giving any explanation. The information gathered from the residents’ prescriptions are personally nonidentifiable data and we are collecting the minimum data set necessary for the study. Data are anonymized from the beginning, coded in the template with the code of the nursing home and a consecutive number for each antibiotic treatment. Individual patient consent is therefore not required.

## Results

The local coordinators in each of the countries have already contacted all the nursing homes participating in the IMAGINE project, with a total of 109 nursing homes. All these nursing homes have already completed the first audit registration. The results of the first audit registrations will be available before the intervention takes place in November 2024. The overall results of the IMAGINE project will be available after summer 2025 and the results will be published by the end of 2025.

## Discussion

We hypothesize that this 2-day multifaceted intervention for health care providers in nursing homes will significantly improve adherence to IPC practices and enhance understanding of when to suspect a UTI. This improved adherence is expected to result in a notable reduction in the rates of inappropriate antibiotic consumption for UTIs. We anticipate that these changes in practices and antibiotic usage will be followed by a subsequent reduction in the incidence of UTIs, as observed during the second registration audit. The overall aim is to enhance the quality of care and patient outcomes through better preventive measures and more judicious use of antibiotics in nursing homes.

The increasing concern about the emergence of AMR in nursing homes necessitates a cohesive and systematic approach to address challenges across very heterogenous settings and diverse health care delivery systems, already under pressure. Additional challenges result in a confluence of risks for colonization or infection with multidrug-resistant pathogens among residents, rendering nursing homes potential reservoirs for AMR [34]. Infections caused by antibiotic-resistant bacteria are associated with a greater need for medical visits; more hospital admissions; higher mortality; and higher economic, intersectoral, and societal costs [35,36]. A recent study tested a multifaceted AMS stewardship intervention in Poland, the Netherlands, Norway, and Sweden and found it effectively and safely reduced UTI antibiotic prescriptions among frail older adults [37]. This study demonstrates that educating care home staff about UTIs and improving their communication with HCPs significantly reduces inappropriate UTI antibiotic prescribing [38,39]. This high use of UTI antibiotics in care homes may be driven by a high prevalence of asymptomatic bacteriuria among residents, which can be as high as 50%, compared to just 4% in older people living independently [40,41]. This is despite no reported benefit from treating asymptomatic bacteriuria with antibiotics. Clinical uncertainty around symptoms such as confusion, falls, and agitation being attributed to UTIs also contributes to the high rate of antibiotic use [42].

Implementing hygiene strategies and reducing the unnecessary prescription of antibiotics has been shown to be the most effective measure to curb the problem of AMR [43,44]. The IMAGINE study aims to address gaps related with AMS and IPC elements through an intervention targeting HCPs to enhance their adherence to good clinical practices. Importantly, this study poses no risks to the HCPs or the residents whose treatment data will be collected. The intervention will use various techniques, including providing feedback to HCPs in nursing homes, conducting follow-up meetings, facilitating discussions on guidelines, offering training courses for HCPs with special focus on when a UTI should be suspected, and distributing informative and educational materials to residents and their relatives.

Furthermore, the IMAGINE study encompasses 8 European countries that exhibit diverse prevalence of antibiotic resistance, distinct cultural backgrounds, varying socioeconomic indices, and different health care systems. This broad scope increases the applicability of the study findings to a wide range of settings. If we find our intervention to be effective, we strongly believe this work has great potential for dissemination. The final results of this quality improvement project will be submitted for publication in a peer-reviewed journal and presented at international scientific conferences. Ultimately, these findings will inform public health interventions aimed at promoting appropriate antibiotic prescribing and implementing effective hygiene strategies in nursing homes.

The use of the Audit Project Odense method has certain limitations. The one notable weakness is the lack of external validity associated with the collected data. Since nursing home HCPs participate voluntarily, their prescribing habits may not accurately represent the average antibiotic use in all nursing homes within their country. Additionally, the nursing homes involved in the audits might exhibit a greater interest in quality improvement and potentially use more rational antibiotic practices compared to nonparticipating nursing homes [45]. Another aspect to consider is the potential influence of conducting an audit on providers’ prescribing behaviors. The HCPs are aware of being observed, which can lead to what is known as the Hawthorne effect. It is important to note that this study is not a clinical trial and lacks a control group, which could be considered a limitation. However, the study will compare the same participants before and after the intervention. Participation in a quality improvement project can pose a barrier due to the required time commitment. While completing a registration takes less than 2 minutes, HCPs need to allocate time for educational courses and other planned activities associated with the intervention. Another weakness is the cross-sectional nature of the Audit Project Odense method. The variables included in the registration chart are aligned with the consultation process, and ideally, the decision to treat should follow the establishment of a diagnosis. However, in practice, diagnostic procedures and treatment decisions are often intertwined [46]. HCPs may determine whether to prescribe a medication concurrently or even prior to establishing a diagnosis. Consequently, clinicians may adjust the diagnosis to align with the treatment decision, potentially introducing a diagnostic misclassification bias. Nevertheless, this potential bias would impact the validity of the diagnosis both before and after the intervention, with minimal likelihood of influencing the intervention’s effectiveness. Due to time constraints in the registration process and limitations on the maximum number of columns in the Audit Project Odense chart, only the typical signs and symptoms of UTIs are collected. This may result in certain limitations, as nonbiomedical factors that could serve as powerful predictors of antibiotic prescriptions are not considered in this study. This restricts the definition of antibiotic appropriateness in our study, as it is based on the collected items in the registration charts.

In conclusion, despite the widespread use of antibiotics and the increasing development of AMR, there have been limited efforts to reduce the occurrence of common infections in nursing homes and improve the diagnosis of UTIs. Moreover, there is a lack of initiatives focused on implementing measures related to hygiene and reducing the inappropriate use of antibiotics in this setting. This protocol has summarized the rationale and design of a before-and-after intervention study aimed at evaluating the impact of a multifaceted intervention on antimicrobial use for UTIs among residents in nursing homes, its appropriateness and the elements of hygiene implemented.

## Appendix

Multimedia Appendix 1 Peer-review Report from the European Commission (EU4 Health Programme).

## Abbreviations

AMR: antimicrobial resistance
AMS: antimicrobial stewardship
HCP: health care professional
IDIAP Jordi Gol: Institut Universitari d’Investigació en Atenció Primària
IMAGINE: improving antibiotic use in nursing homes by infection prevention and control and antibiotic stewardship
IPC: infection prevention and control
UTI: urinary tract infection

## Data Availability

The data sets generated or analyzed during this study are available from the corresponding author on reasonable request.
