# Supplementary material for: Improving Antibiotic Use in Nursing Homes by Infection Prevention and Control and Antibiotic Stewardship (IMAGINE): Protocol for a Before-and-After Intervention and Implementation Study
Source: JMIR Res Protoc. 2024 Sep 16;13:e60099. doi: 10.2196/60099 (PMC11444125; doi:10.2196/60099)
Supplement: Multimedia Appendix 1 [file resprot_v13i1e60099_app1.pdf]

Proposal Evaluation Form

EUROPEAN COMMISSION

EU4 Health Programme (EU4H)

Evaluation Summary Report

**Call:**	EU4H-2021-PJ2
**Type of action:**	EU4H-PJG
**Proposal number:**	101079838
**Proposal acronym:**	IMAGINE
**Duration (months):**	36
**Proposal title:**	Improving antibiotic use in long term care facilities by infection prevention and control and antibiotic stewardship
**Activity:**	EU4H-2021-PJ-14

N.	Proposer name	Country	Total Cost	%	Grant Requested	%
1	INSTITUT CATALA DE LA SALUT	ES	211,860	10.28%	169,488	10.28%
2	REGION HOVEDSTADEN	DK	386,341.69	18.74%	309,073.35	18.74%
3	FORSKNINGSENHEDEN FOR ALMEN PRAKSIS	DK	229,301	11.12%	183,440.8	11.12%
4	FUNDACIO INSTITUT UNIVERSITARI PERA LA RECERCA A L'ATENCIO PRIMARIA DE SALUT JORDI GOL I GURINA	ES	278,681.5	13.52%	222,945.2	13.52%
5	PANEPISTIMIO KRITIS	EL	104,860	5.09%	83,888	5.09%
6	UNIVERSYTET MEDYCZNY W LODZI.	PL	104,860	5.09%	83,888	5.09%
7	UNIVERZA V LJUBLJANI	SI	104,860	5.09%	83,888	5.09%
8	SLOVENSKA ZDRAVOTNICKA UNIVERZITA V BRATISLAVE	SK	101,650	4.93%	81,320	4.93%
9	MANO SEIMOS GYDYTOJAS	LT	104,860	5.09%	83,888	5.09%
10	Oszido Nonprofit Kft	HU	100,259	4.86%	80,207.2	4.86%
11	UNIVERSIDAD DE LAS PALMAS DE GRAN CANARIA	ES	105,823	5.13%	84,658.4	5.13%
12	SOCIEDAD ESPANOLA DE MEDICINA DE FAMILIA Y COMUNITARIA	ES	86,250.56	4.18%	69,000.44	4.18%
13	EUROPEAN UNION GERIATRIC MEDICINE SOCIETY AISBL	BE	49,220	2.39%	39,376	2.39%
14	E.C.H.O. European Confederation of Care Home Organisations	BE	49,220	2.39%	39,376	2.39%
15	EUROPAISCHE VEREINIGUNG FUR KLINISCHE PHARMAKOLOGIE UND THERAPIE EV	DE	43,228	2.10%	34,582.4	2.10%
	Total:		2,061,274.75		1,649,019.79	

Abstract:
Excessive and inappropriate use of antibiotics is the main driver of antimicrobial resistance, which is particularly problematic, especially in frail older people who are susceptible to frequent and severe infections. A lack of infection prevention and control (IPC) and inappropriate diagnosis leads to antimicrobial overprescribing in long-term care facilities (LTCF). In this setting, health care-associated infections, mainly urinary tract infections (UTI), some of which caused by resistant bacteria, are common but could be reduced through antimicrobial stewardship (AMS) and improved IPC measures, but the implementation hugely vary across countries.
The aim of this project is to increase the prevention of infections – mainly UTIs –and reduce antibiotic inappropriateness for these infections by implementing a multifaceted intervention targeting health care professionals (HCP) in LTCFs including in particular nursing homes. The project applies the Audit Project Odense method of audit and feed-back before and after this intervention.
We will include 10 LTCFs in each of the eight countries participating in the project, seven of which are low-income member states (LIMS) countries (Greece, Hungary, Lithuania, Poland, Slovakia, Slovenia, and Spain) and Denmark.
For three months, the HCPs in each LTCF will register information about UTIs and other infections in the LCTF, as well as the IPC measures. HCPs will then receive a multifaceted intervention. The registration will be repeated after one year of the first registration. We aim to include the LCTF staff, both the personnel responsible for nursing, such as registered nurses, healthcare assistants and helpers and the doctors responsible for diagnostics and prescribing of antibiotics.

Evaluation Summary Report

Evaluation Result

Total score: 97.00 (Threshold: 70)

Criterion 1 - Relevance

Score: **30.00** (Threshold: 21/30.00 , Weight: -)

The detailed criteria are set out in the call conditions (see Call document).

The rationale of the project's background information is well described. The background is to reduce excessive antibiotic use since this leads to the development and spread of antimicrobial resistance, particularly since antibiotics are overly used in long-term care facilities.

The rationale of the project is excellent since infections are very prevalent amongst elderly people, due to a number of factors such as a weaker immune system and intense and long use of devices such as indwelling urinary catheters.

The project's contribution to the priorities of the call include pursuing better prevention and control of antimicrobial resistance through the understanding of the use of antibiotics and long term facilities to reduce the use of antibiotics. This will lead to good practices in infection prevention and control. There is not any national surveillance system in the EU for the surveillance of such infections in long-term care facilities.

The objectives of the project are concrete, clearly outlined, ambitious and realistic within the duration of the work. The specific objectives of the call include tackling the over use of antibiotic in suspected urinary tract infections in long term care facilities, since in Europe this is leading to the development and spread of multi drug resistant uro-pathogens. The objectives correspond to the EU4Health program priorities, e.g. a rational usage of antimicrobial medications and a reduction of antimicrobial resistance. The main and secondary objectives are clear, measurable, realistic and achievable within the duration of the project.

The project's aspects and contributions to the results of past activities carried out in the field are well described. The project builds on from the past activities of previously awarded EU grants. Most of the partners that participated in previously awarded EU grants are also participating in this project. There is also a number of nursing homes used in a number of countries that have been used in previous grants, but there will be more quantitative and additional studies bringing added value to the project.

This proposal is innovative because it includes the infection prevention and control elements that have not been considered in previous proposals including urinary tract and related infections in the elderly. Health care professionals in all long-term care facilities will take part in the project implementation from the beginning until the end of the project and this will bring additional value for outcomes and sustainability.

The European dimension of the activities is clearly laid out and a number of EU countries will benefit from the project results either directly or indirectly. The target countries for training and the activities where this will take place include Denmark, Spain, Greece, Poland, Slovenia, Slovakia, Lithuania, and Hungary, and seven are low-income countries. The potential to develop cross border cooperation is clearly evidenced because the guidelines and implementation of the infection prevention control measures will provide public health policy makers with the ability to assess the likely impact of the implementation of the objectives used from this project.

Criterion 2.1 Quality — Project design and implementation

Score: **29.00** (Threshold: 21/30.00 , Weight: -)

The detailed criteria are set out in the call conditions (see Call document).

The project's approach and methodology are very well outlined, and described pertaining to the details of why these are the most suitable and innovative to achieve the project's objectives. The implementation includes the education of nursing and medical staff, audits of antimicrobial use, feedback to the prescribers and targeting identified areas of antimicrobial overuse and misuse. The communication issues between nursing home teams and residents will also be evaluated, as well the communication pathway between the nursing homes and doctors.

Local experts and trainers will help to acquire all necessary equipment for better results during implementation of the tools. Simple charts will be used for registration of symptoms, and will provide a real tool for accurate collection data for analysis. A concise work plan including several work packages, realistic and achievable milestones, and deliverables is very clearly explained.

Particular attention is given by the consortium to provide with indicators, e.g. capacity building activities, as the proposal includes expected targets for the organisation of training courses (80), the trained specialists (320), the nursing homes involved (80), and the outreach events (8 workshops). The total number of registrations to be carried out in the project is estimated to be at least 7600.

As for the best practices, the project aims to trigger change in innovation by achieving significantly higher stakeholder knowledge and implementation of infection prevention control and antimicrobial stewardship in nursing homes by implementing research identified best practices with training in hygiene activities. The verification measures are very well planned to ensure that the project implementation is of high quality and will be completed in time.

The evaluation methods and indicators (quantitative and qualitative) to monitor and verify the outreach and coverage of the activities and results are relevant, realistic, and measurable. Process evaluation and evaluation of results are well explained. With the use of participatory action research and a proven methodology called Audit Project Odense, and with feedback and discussion of results before and after a multifaceted intervention for nursing home staff, the implementation of these measures will be improved. Realistic measures of impact indicators are very clearly defined.

The consortium will bring together the necessary expertise through well organised work packages. The consortium members are experienced and complement one another's areas. The consortium partners have collaborated on previous related projects, and they have experience in managing European and national projects and day-to-day management of research projects, especially those focused on infections and antimicrobial stewardship, cooperation with nursing homes and employment implementation of measures to prevent the spread of infections.

The participants include governmental workers, hospital owners, research organisations, universities, non-profit organisations and nursing homes. Cooperation with other international and regional organizations and associations are already established.

The cost-effectiveness and financial management of the project are well described and credible. All partners are legal entities with relevant experience in financing procedures and projects, and will provide financial monitoring and updates.

All tools will be prepared in the partners own languages, which will guarantee better motivation of participants and successful further dissemination of positive results in long-term period. Stakeholders will be included including decision makers health authorities, nursing home owners and final users, and these will be invited to project meetings.

The specific level action indicators mentioned in the call text are missing and should be added during the grant agreement preparation.

Criterion 2.2 Quality — Project team and cooperation arrangements

Score: **29.00** (Threshold: 21/30.00 , Weight: -)

The detailed criteria are set out in the call conditions (see Call document).

The consortium participants, including the project coordinator and work package leader institutions, are complementary and their roles are well outlined and described with regard to their tasks and contributions for the smooth implementation of the project. The management structure includes a coordinator of the project and a project manager.

Using their different expertise, the consortium members will carry out the project management, ethical management, project administration, training resources, context analysis, dissemination and communication required. In addition, feedback reports and meetings, recruitment of health professionals, audit registration from the nursing homes, or the health professionals, health economics and upscaling, statistical analysis, recommendation process evaluations during the project engagement with stakeholders, and dissemination to healthcare providers will be carried out.

The decision-making process is well described. Decisions will be taken by consensus, but if that cannot be achieved within a reasonable time, decisions will be made by the management structure. Work package leaders will take decisions at work package level but if technical decisions have consequences for other work packages, then the work package leader will take these decisions after consulting with the coordinator and other work package leaders. The steering board will take final decisions at project level. To ensure effective planning and control, a project management system that has already been used in previous EU projects will be integrated. Efficient and timely communication and robust administration will be made in line with all legal requirements and good practices.

The methods to ensure quality control include having procedures for quality assessment and indicators for the measurement of the success of the quality and performance of the project. The proposal includes a work package that has a dedicated task and deliverable to evaluate the quality and progress of the project. Evaluation reports will be prepared after each partner meeting, and all participants will be invited to provide their feedback. The feedback reports will be used to adjust the project management procedures if needed.

Evaluation will be carried out to determine the short-term project activities on the results and a set of indicators will be defined and collected according to the evaluation plan. The indicators will be discussed, and agreed among all the relevant stakeholders. Evaluation reports will be prepared after each partner meeting. Alongside the quantification of the direct outcomes of the project, the consortium will design and implement a model to estimate a scale up scenario for the execution of the results found in this project. This evaluation will include clinical and economic evaluation of the data collected within the project, which will be available for a detailed social return on investment analysis.

The consortium has adopted measures and objectives to ensure the most cost effective manner in accordance with the rules necessary for the implementation of the actions. The competencies required for the execution of the project are well covered by the partners. The governance structure and procedures are prepared and established. The main responsibilities are divided among partners and coordinators. Most experts have experience in infection control processes, teaching, and research. Partners' collaboration and participation in previous/ongoing EU-funded projects are well documented.

The total budget is calculated based on partner budgets to ensure that the results on objectives will be achieved in the most cost effective way.

Criterion 3 - Impact

Score: **9.00** (Threshold: 7/10.00 , Weight: -)

The detailed criteria are set out in the call conditions (see Call document).

The short, medium and long-term effects of the project are well defined and credible and include the training of 320 healthcare professionals from eight countries. The long-term effects of the project are foreseen and financial savings of health systems are calculated. The healthcare professionals will inform their colleagues and be supported by communication through professional networks to disseminate the results and experiences. The healthcare professionals that are trained will train at least 30 other healthcare professionals in the same setting in each country. The long-term impact will be upscaling of the intervention materials into different languages and these will be used for training staff in nursing homes in other European countries.

The target groups and how they will benefit concretely from the project are clearly addressed. The target groups are the elderly in long-term care facilities to reduce antimicrobial resistance through the over prescription of antibiotics due to misdiagnosis of urinary tract infections. Stakeholders include policy makers, long-term care facility staff residents and their relatives, healthcare professionals and will also involve hospitals and primary care and professional organisations.

The change/innovation that the project aims to trigger is well described. It is the first project to seek both implementation activities together with comprehensive recent research into failings of past intervention strategies. In addition, potential new routes will be taken forward into a series of actions which can be adapted to nursing homes in every region and country.

The communication and dissemination activities which are planned to promote the results will be addressed to both professional groups and the general public. There will be a project website and social media channels. Three target groups are foreseen for managing the dissemination of the results of the project through local and international organizations. The final Conference will be provided openly for all stakeholders. The impact and effectiveness of the communication to dissemination activities will be continuously monitored using quality quantitative performance indicators including outreach data and engagement indicators.

The sustainability of the project after the EU funding ends is very well explained as are the synergies/complementarities with other activities. Follow-up of the project after the EU funding ends will be carried out by establishment of cross-sectional antimicrobial resistance networks and national nursing training projects which will be relevant to a number of nursing homes in the countries involved and will also be applicable to other EU countries.

Scope of the proposal

Status: **Yes**

Comments (in case the proposal is out of scope)

Not provided

Exceptional funding

Entities from countries mentioned in the work programme (if any) are only exceptionally eligible, if the granting authority

considers their participation essential for the implementation of the action.
Please list the concerned applicants and requested grant amount and explain the reasons why.

Based on the information provided, the following participants should receive exceptional funding:

Not provided

Based on the information provided, the following participants should NOT receive exceptional funding:

Not provided

Higher funding rate (priority actions 80%) (if applicable)

Status: **Yes**

If YES, is the action an 'action with exceptional utility' as defined in the Call document

Yes

Give reasons:

The criteria of budget distribution set out in the call document for the call for action grants EU4H-2021-PJ-2 are fulfilled.
a) at least 30 % of the budget of the proposed action is allocated to Member States whose GNI per inhabitant is less than 90 % of the Union average; or
b) bodies from at least 14 participating Member States participate in the action, of which at least four are Member States whose GNI per inhabitant is less than 90 % of the Union average.
Given this compliance with an action with exceptional utility, the higher funding rate is recommended.

Overall comments

Not provided

Digitally sealed by the European Commission

Date: 2022.07.08 17:25:35 CEST

This electronic receipt is a digitally signed version of the document submitted by your organisation. Both the content of the document and a set of metadata have been digitally sealed.

This digital signature mechanism, using a public-private key pair mechanism, uniquely binds this eReceipt to the modules of the Funding & Tenders Portal of the European Commission, to the transaction for which it was generated and ensures its full integrity. Therefore a complete digitally signed trail of the transaction is available both for your organisation and for the issuer of the eReceipt.

Any attempt to modify the content will lead to a break of the integrity of the electronic signature, which can be verified at any time by clicking on the eReceipt validation symbol.

More info about eReceipts can be found in the FAQ page of the Funding & Tenders Portal.

(https://ec.europa.eu/info/funding-tenders/opportunities/portal/screen/support/faq)
